# Emergency surgery in the era of artificial intelligence: ChatGPT could be the doctor’s right-hand man

**DOI:** 10.1097/JS9.0000000000000410

**Published:** 2023-04-19

**Authors:** Kunming Cheng, Zhiyong Li, Qiang Guo, Zaijie Sun, Haiyang Wu, Cheng Li

**Affiliations:** a Department of Intensive Care Unit, The Second Affiliated Hospital of Zhengzhou University, Zhengzhou, Henan; b Department of Orthopedics, Baodi Clinical College of Tianjin Medical University, Tianjin; c Department of Orthopaedic Surgery, Xiangyang Central Hospital, Affiliated Hospital of Hubei University of Arts and Science, Xiangyang; d Clinical College of Neurology, Neurosurgery and Neurorehabilitation, Tianjin Medical University, Tianjin; e Department of Orthopaedic Surgery, Beijing Jishuitan Hospital, Fourth Clinical College of Peking University, Beijing, People’s Republic of China; f Duke Molecular Physiology Institute, Duke University School of Medicine, Durham, North Carolina, USA

*Dear Editor,*

Emergency surgery refers to an operation that needs to be performed immediately due to a life-threatening condition. This kind of emergency intervention is common in the field of surgery, especially in general surgery, neurosurgery, and orthopedics. With the rapid development of artificial intelligence (AI), approaches that combine AI with medicine are flowering everywhere across multiple medical fields including emergency surgery^1^. Recently, ChatGPT, developed by OpenAI, an AI-based natural language processing tool that allows human-like conversations with the chatbot, has recently gained tremendous attention in the public and academia. It is fine-tuned from a model in the GPT-3.5 series. Writing scientific papers, code modification, and passing the United States Medical Licensing Examinations (USMLE), ChatGPT continues to demonstrate magical abilities beyond imagination in various fields. In addition, a growing number of studies have explored the potential applications of ChatGPT in various medical disciplines, such as endocrinology, radiology, hepatology, obstetrics and gynecology, and so on^2–4^. On 14 March 2023, OpenAI announced the release of the new and vastly improved GPT-4. Compared with GPT-3.5, the most remarkable characteristic of this new version is its ability to handle images and text, which could further revolutionize the way humans interact with computers^5,6^. However, despite this, no study has yet analyzed the potential applications of ChatGPT, especially GPT-4, in emergency surgery. In view of this, in the present study, we will talk about the various ways in which ChatGPT could serve as the doctor’s right-hand man in the field of emergency surgery.

## Rapid assessment and severity classification

**Figure 1:**
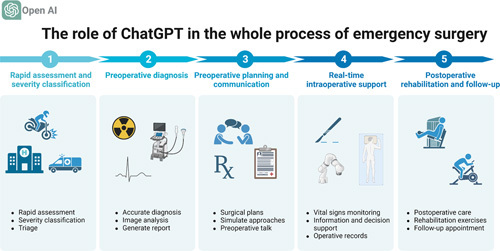
The potential role of ChatGPT in the whole process of emergency surgery (created by BioRender).

As a powerful tool of AI, ChatGPT could provide possible diagnostic suggestions within seconds after inputting patients’ information such as symptoms, past medical history, and current condition. Meanwhile, as a supportive role, ChatGPT is able to offer basic information on first aid techniques for various medical conditions and also assist professionals in conducting information retrieval for the best practices and guidelines. In addition, another potential application of ChatGPT within emergency medicine is in triage, which help prioritize patients according to the urgency of their need for medical attention. All in all, with the aid of ChatGPT, the prehospital emergency session will become more efficient. This could reduce the time taken to initiate life-saving procedures and thereby be used by the surgeon to create an effective emergency surgery plan (Fig. 1).

## Assisting in preoperative diagnosis

ChatGPT’s advanced natural language understanding and generation capabilities offer potential support for analyzing large volumes of medical data to identify complex patterns and relationships. And also help emergency surgeons better understand a patient’s medical history, symptoms, and laboratory results, thus contributing to a more accurate diagnosis. Additionally, as GPT-4 started to accept image input, if the application programming interface of ChatGPT accessed the clinical picture archiving and communication systems, it could automatically generate a simplified report for emergency physicians, which could further shorten the waiting time for surgery^5^. Moreover, ChatGPT has the ability to learn from new databases, which means it can continually update the knowledge base, keeping pace with the latest medical advancements. Thus, it may provide better-informed treatment strategies.

## Preoperative planning and communication

Firstly, AI-based algorithms learn from historical patient data; ChatGPT has huge potential to be used to generate surgical plans and simulate different approaches, which enable emergency surgeons to optimize surgical techniques, identify potential complications, and improve the success rate of surgery. Secondly, for young surgeons without enough experience, ChatGPT could serve as a virtual mentor, helping them increase their skills and confidence in handling complex cases. Thirdly, as an AI-based amplopedia, ChatGPT could provide detailed information about various surgical procedures and their indications, contraindications, risks, and expected outcomes. Take preoperative talk as an example; it is an important step to allow patients to make informed choices about the appropriateness of surgery and tolerance of the risks and treatment costs brought by the surgery. We have attempted to use GPT-4 to generate the surgical risk checklist for two different groups of emergency patients, whose only difference is one comorbid with hypertension and diabetes mellitus. The results of this test are shown in Supplementary File 1, Supplemental Digital Content 1, http://links.lww.com/JS9/A379. As can be seen, ChatGPT is able to identify the additional surgical risk brought by hypertension and diabetes mellitus.

## Real-time intraoperative support

In our understanding, ChatGPT could provide intraoperative support for emergency surgeons in the following manner: *Vital signs monitoring*: Although ChatGPT may not be able to directly monitor patients’vital signs, it could support as the early warning system to give the reminder and suggestions when the unexpected situation happened. *Information and decision support*: During surgery, unforeseen conditions may occur that require surgeons’ immediate action. ChatGPT could help surgical teams access real-time guidance, case studies, or reference materials based on a comprehensive understanding of the patient’s condition, which may be useful for surgeons addressing unexpected challenges under stress. Meanwhile, based on the latest guidelines provided by ChatGPT, this facilitated more informed decision-making procedures that adhere to the most current standards during the operation. *Operative records*: With the help of ChatGPT, intraoperative data could be recorded in real-time, which is convenient for postoperative medical records and further use in scientific research.

## Assisting in postoperative rehabilitation and follow-up

Similar to the above-mentioned, ChatGPT also could provide general information on postoperative care, rehabilitation exercises, and guidelines from relevant medical societies, ensuring that postoperative rehabilitation follows the most current standards. ChatGPT could become a resource for patients to acquire out-of-hospital instruction. Take acute appendicitis as an example; when we ask GPT-4 how long the patients can go back to work or exercise after an emergency appendectomy, the answer is professional and meticulous (Supplementary File 2, Supplemental Digital Content 2, http://links.lww.com/JS9/A380). ChatGPT even considers different surgical approaches, such as laparoscopic or open appendectomy, should have different guidelines. Meanwhile, by providing reminders for rehabilitation exercises, medication schedules, and follow-up appointments, ChatGPT may be used to monitor the recovery progress. More importantly, by analyzing the follow-up data, ChatGPT could help identify early complications after emergency surgery, prompting patients to acquire timely secondary intervention when necessary.

## Outlook

In this study, we discussed various ways in which ChatGPT could serve as an indispensable tool for emergency surgical interventions. Overall, with its advanced natural language understanding and generation capabilities, ChatGPT could assist the whole process of emergency surgery including rapid assessment and severity classification, preoperative diagnosis, preoperative planning and communication, real-time intraoperative support, and postoperative rehabilitation and follow-up. In our opinion, the advent of ChatGPT offers promising opportunities to revolutionize emergency surgery. Meanwhile, as the technology continues to evolve and mature, we anticipate that ChatGPT and similar AI-based tools will become increasingly important in the field of emergency surgery. And could serve as the right-hand man for emergency surgeons.

## Ethical approval

This study does not include any individual-level data and thus does not require any ethical approval.

## Sources of funding

This study is supported by China Postdoctoral Science Foundation (2022M720385) and Beijing JST Research Funding (YGQ-202313).

## Author contribution

K.C.: methodology, data curation, formal analysis, resources, investigation, writing – original draft, and writing – review and editing; Z.L. and Z.S.: conceptualization, methodology, data curation; Q.G.: investigation, writing – original draft, and writing – review and editing; H.W.: conceptualization, methodology, data curation, formal analysis, resources, investigation, writing – original draft, and writing – review and editing; C.L.: conceptualization, formal analysis, resources, investigation, and writing – review and editing.

## Conflicts of interest disclosure

The authors declare no conflicts of interest.

## Research registration unique identifying number (UIN)

1. Name of the registry: not applicable.
2. Unique identifying number or registration ID: not applicable.
3. Hyperlink to your specific registration (must be publicly accessible and will be checked): not applicable.

## Guarantor

Zaijie Sun, Haiyang Wu, and Cheng Li.

## Data availability statement

The data underlying this article will be shared by the corresponding author on reasonable request.

## Supplementary Material
