# Supplementary figures and images for: Emergency surgery in the era of artificial intelligence: ChatGPT could be the doctor’s right-hand man
Source: Int J Surg. 2023 Apr 19;109(6):1816–8. doi: 10.1097/JS9.0000000000000410 (PMC10389530; doi:10.1097/JS9.0000000000000410)

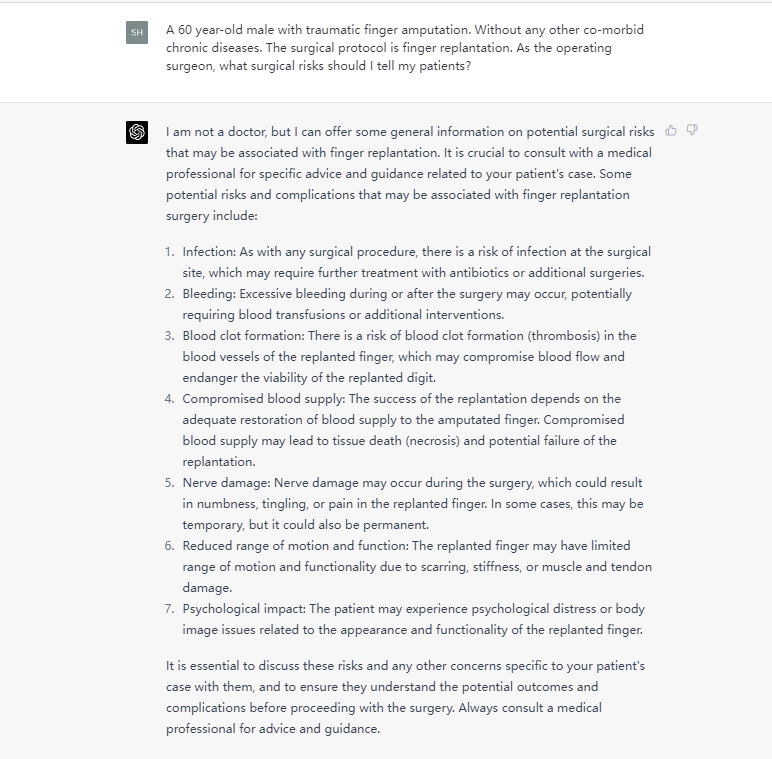


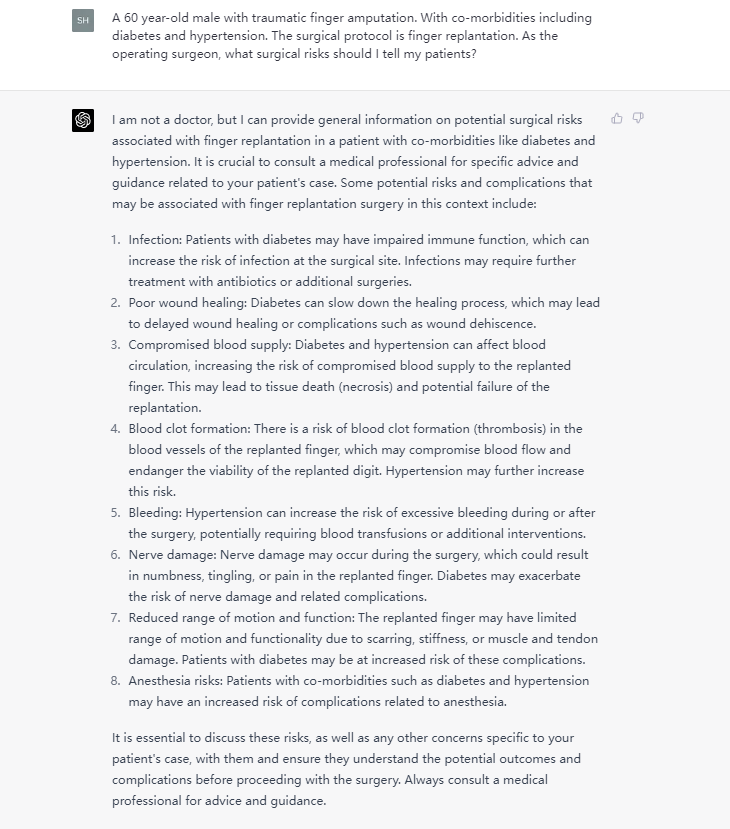

Supplement: Supplementary file 1 [file js9-109-1816-s001.docx]

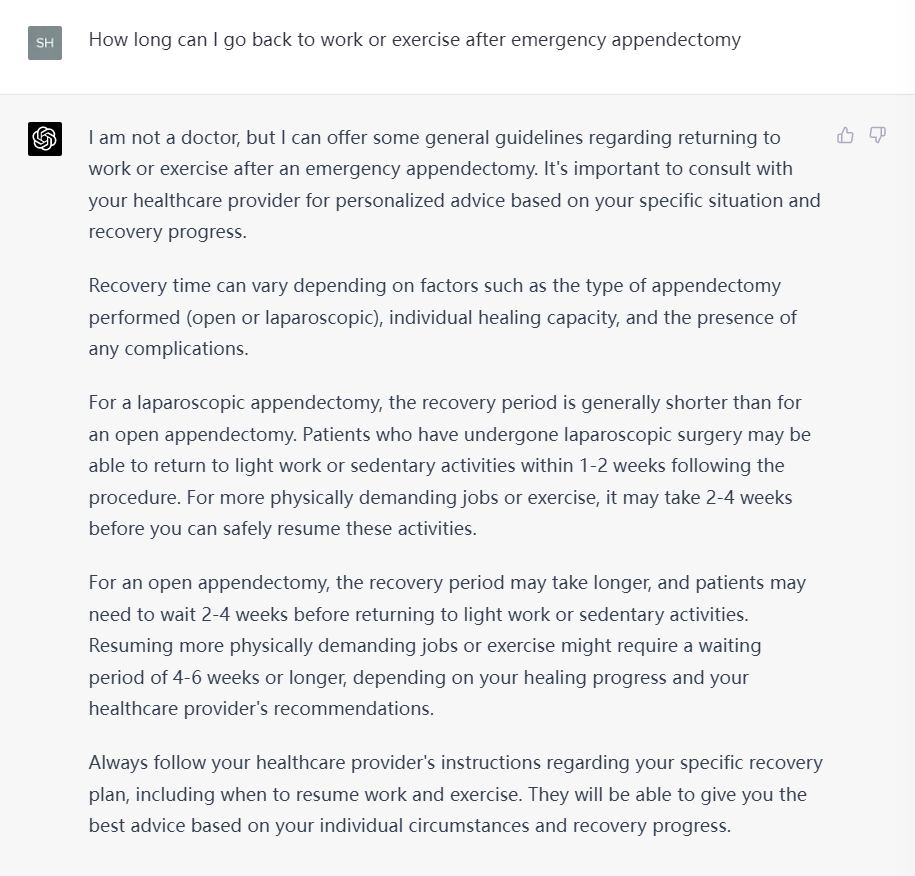

Supplement: Supplementary file 2 [file js9-109-1816-s002.docx]
